# Factors associated with TBS worse than BMD in non-osteoporotic elderly population: Bushehr elderly health program

**DOI:** 10.1186/s12877-021-02375-8

**Published:** 2021-07-27

**Authors:** Nekoo Panahi, Afshin Ostovar, Noushin Fahimfar, Hamid Reza Aghaei Meybodi, Safoora Gharibzadeh, Babak Arjmand, Mahnaz Sanjari, Kazem Khalagi, Ramin Heshmat, Iraj Nabipour, Akbar Soltani, Bagher Larijani

**Affiliations:** 1 Osteoporosis Research Center, Endocrinology and Metabolism Clinical Sciences Institute, Tehran University of Medical Sciences, Tehran, Iran; 2 Endocrinology and Metabolism Research Center, Endocrinology and Metabolism Clinical Sciences Institute, Tehran University of Medical Sciences, Tehran, Iran; 3 Evidence Based Medicine Research Center, Endocrinology and Metabolism Clinical Sciences Institute, Tehran University of Medical Sciences, Tehran, Iran; 4 Department of Epidemiology and Biostatistics, Pasteur Institute of Iran, Tehran, Iran; 5 Metabolomics and Genomics Research Center, Endocrinology and Metabolism Molecular-Cellular Sciences Institute, Tehran University of Medical Sciences, Tehran, Iran; 6 Chronic Diseases Research Center, Endocrinology and Metabolism Population Sciences Institute, Tehran University of Medical Sciences, Tehran, Iran; 7 The Persian Gulf Marine Biotechnology Research Center, The Persian Gulf Biomedical Sciences Research Institute, Bushehr University of Medical Sciences, Bushehr, Iran

**Keywords:** Bone mineral density, Trabecular bone score, Osteoporosis, Discordance, Men, Women, Aging, Iran

## Abstract

**Background:**

Bone mineral density (BMD) and trabecular bone score (TBS) are moderately correlated. TBS is sometimes used as an adjuvant to BMD in the fracture risk assessment. Some individuals with normal BMD or osteopenia, have more degraded TBS. We aimed to identify factors associated with TBS worse than BMD in the non-osteoporotic elderly population.

**Methods:**

The study subjects were selected from 2384 women and men aged ≥60 years participating in the second stage of the Bushehr Elderly Health program, a population-based prospective cohort study in Iran. The BMDs of different sites and the lumbar spine texture were measured using dual-energy X-ray absorptiometry and the TBS algorithm, respectively. Subjects were categorized based on their BMD and TBS status. Logistic regression was performed to identify the factors associated with “TBS worse than BMD” in non-osteoporotic individuals.

**Results:**

Of 1335 participants included in the study, 112 of 457 women, and 54 of 878 men had worse TBS than BMD. In multivariable analysis, TBS worse than BMD in women was statistically significantly associated with years since menopause (OR: 1.04 (1.00–1.07)) and waist circumference (OR: 1.09 (1.05–1.14)). However, in men, the condition was statistically significantly associated with waist circumference (OR: 1.10 (1.03–1.17)), current smoking (OR: 2.54 (1.10–5.84)), and HDL-C (OR: 1.03 (1.00–1.06)).

**Conclusion:**

The results of the study show that higher waist circumference is associated with more degraded TBS than BMD in both men and women. Years passed since menopause and current smoking, respectively in women and men, were associated with more degraded TBS. Considering TBS values in older individuals with higher waist circumference, or a history of smoking despite normal BMDs might help more accurate assessment of bone health. However, further studies are required to confirm the benefit.

## Introduction

The diagnosis of osteoporosis is most commonly based on bone mineral density (BMD) using dual-energy X-ray absorptiometry (DXA). However, bone strength is dependent on both bone mass and bone quality (bone geometry, micro-architecture, micro-damage, mineralization, and turnover) [1], as evidenced by the occurrence of more fragility fractures in normal BMD or osteopenic individuals than in osteoporotic ones [2, 3]. Fractures associated with osteoporosis impose a great burden, both short-term and long-term [4]. The condition is preventable and treatable, however, only a small proportion of those at increased risk for fracture is evaluated and treated. Many elderly men, as well as women, are left under-screened and left undiagnosed and undertreated [5].

Trabecular bone score (TBS) as a surrogate of bone microstructure and bone quality, can be measured using TBS iNsight software (version 2.2; Medimaps Group, Plan-les-Ouates, Switzerland) which can be applied prospectively or retrospectively to any standard DXA images obtained with the use of modern fan-beam densitometers [6]. An elevated TBS corresponds to a stronger trabecular structure, while a low TBS is related to a weak fracture-prone bone [6, 7].

BMD and TBS are moderately correlated [8, 9]. TBS can be used as a complement to the BMD in fracture risk assessment [10–12] and is partially independent of BMD with different predicting factors. Different conditions including but not limited to glucocorticoid excess, primary hyperparathyroidism, higher free thyroid hormone, diabetes mellitus type 2, androgen-deficiency, chronic kidney disease, autoimmune disorders like rheumatoid arthritis and ankylosing spondylitis, obesity, and smoking are associated with a low TBS and increased fracture risk, independent of BMD [6, 13–19]. According to a large sample size cohort using Manitoba BMD Registry, the investigators evaluated the effect of TBS after a minimum of 5 years of observation in 37,176 subjects and they found that TBS could predict major osteoporotic fractures and/or hip fracture overall, and several of the considered clinical variables showed significant interactions with TBS. TBS-adjusted fracture risk assessment tool (FRAX) resulted in significant risk reclassification and/or improved fracture risk classification for most of the clinical variables considered, especially in women close to the intervention threshold of BMD [13]. According to the International Society for Clinical Densitometry Official Positions (ISCD) [20], TBS is associated with osteoporotic fractures in postmenopausal women and men older than 50 years; it can be used in association with the FRAX [10] and BMD, but not in isolation, to adjust the fracture risk assessment.

Most of the fragility fractures occur in non-osteoporotic individuals according to their BMD levels, and consideration of non-BMD factors which contribute to the bone quality seems mandatory in the at-risk population. Therefore, we aimed to identify elderly women and men with discordant BMD and TBS categories and to determine factors associated with more degraded TBS than BMD in non-osteoporotic elderly Iranian women and men.

## Methods

### Study population

The present study was undertaken using the data of the second stage of the Bushehr Elderly Health (BEH) program. The rationale and design of the original study are described previously [21, 22]. The BEH program, as a population-based prospective cohort study, is being conducted in Bushehr, a southern province of Iran to investigate the prevalence of non-communicable diseases and the contributing risk factors. Elderly women and men aged ≥60 years with adequate physical and mental abilities were enrolled in the cohort. To estimate the prevalence of musculoskeletal disorders and their clinical consequences, the second stage began in October of 2015. The study was approved by the Research Ethics Committee of both Bushehr University of Medical Sciences and Endocrinology and Metabolism Research Institute associated with Tehran University of Medical Sciences. After an explanation of the study objectives and protocols, written informed consent was obtained from all participants.

### Measurements and definition

Medical history was obtained through an interview by trained nurses based on a 162-item questionnaire, including lifestyle factors, such as smoking, physical activity, supplements, and nutritional status. Anthropometric measurements were taken with shoes removed and the participants wearing light clothing. Height and weight were measured with a fixed stadiometer and a digital scale according to the standard protocol. A flexible, circumference measuring tape was used to measure waist circumference (WC) at a point midway between the iliac crest and the lowest rib in a standing position as described in the protocol [21]. An overnight fasting venous blood sample was obtained for every participant for biochemical measurements. Among all, fasting plasma glucose, lipid profile, and creatinine were measured at baseline and their methods of measurement are described elsewhere [21].

Type 2 diabetes mellitus was ascertained among participants who had fasting plasma glucose ≥126 mg/dl, HbA1C ≥ 6.5 or taking anti-diabetic medication. Participants who were smoking cigarettes daily or occasionally and those who used hookah or pipes were classified as current smokers. Physical activity (low or active) was determined according to the physical activity level. Physical activity level in 24 h of sports, work and leisure time on an average weekday was assessed using a valid self-report questionnaire [23], and the intensity of every specific activity was expressed in metabolic equivalents. Participants were categorized into low active or active groups using the cutoff value of 1.6 for physical activity level, which is calculated by dividing total daily energy expenditure by the basal energy expenditure [24]. The total amount of each specific food and beverage consumed was captured by an expert nutritionist. Standard reference tables were used to convert household portions to grams. Data were entered into the nutritionist IV package modified for Iranian foods to obtain daily energy, nutrient intakes and servings of foods consumed. For mixed dishes, food groups and nutrients were calculated according to their ingredients. Calcium intake was categorized as high > 1000 mg/day, moderate 500–1000 mg/day, and low < 500 mg/day. Fracture history was determined by the history of fractures with minor trauma after age 45 years.

### BMD and TBS

BMD for each participant was measured using DXA (Discovery Wi Bone Densitometer; Hologic, Bedford, MA, USA) in a correct position, performed by a trained operator. TBS iNsight software (version 2.2; Medimaps Group, Plan-les-Ouates, Switzerland) was used for TBS measurements. We included individuals with a BMI range of 15 to 37 Kg/m^2^.

We categorized the subjects based on the lowest DXA T-score of the lumbar spine, femoral neck, or total hip according to the National Osteoporosis Foundation [25] and the ISCD classification [26] as follows:

- Group 1, with the lowest T-score ≥ − 1.0 as normal;
- Group 2, with the − 2.5 < lowest T-score < − 1.0 as Osteopenia;
- Group 3, with the Lowest T-score ≤ − 2.5 as Osteoporosis.

TBS classification was performed based on cut-off points suggested in a meta-analysis [10] as following:

- Group 1, with TBS > 1.31 as normal;
- Group 2, with TBS 1.23–1.31 as partially degraded;
- Group 3, with TBS < 1.23 as degraded.

### Identifying non-osteoporotic subjects with more degraded TBS compared to BMD

In our analysis, we excluded the subjects with the diagnosis of osteoporosis based on the BMD of the 3 sites. Further, we categorized the non-osteoporotic subjects into two groups as follows:

- *Discordant*, defined as those with *“TBS worse than BMD”* including subjects with degraded TBS/normal BMD, degraded TBS/osteopenia, and partially degraded TBS/normal BMD;
- *Others*, including subjects with normal BMD/normal TBS, osteopenia/normal TBS, and osteopenia/partially degraded TBS.

### Statistical analysis

We stratified the participants by sex. The Shapiro-Wilk test was used to check normality. Data with normal distribution were expressed as mean (± SD), while skewed variables were expressed as median and interquartile range. To compare different variables between groups, we used the independent t-test (normal variables), Mann-Whitney U test (skewed variables), and chi-square test (categorical variables).

Logistic regression was performed to identify the factors associated with discordance defined as “TBS worse than BMD” in non-osteoporotic elderly individuals. Age, years since menopause in women, body mass index (BMI), WC, diabetes mellitus, smoking status, physical activity, vitamin D supplement, calcium supplement, and daily calcium intake were considered as potentially influential factors. We also considered lipid profile including total cholesterol (TC), triglyceride (TG), high-density lipoprotein cholesterol (HDL-C), low-density lipoprotein cholesterol (LDL-C) because there is likely to be confounding of lipid parameters with WC, BMI, and/or diabetes status. Variables having a significant univariable test at 0.2 level were selected as potential confounders and used in the multivariable analysis.

The area under a receiver operating characteristic (ROC) curve (AUC) was used to evaluate the performance of WC as a predictor of discordance (defined as TBS worse than BMD) in non-osteoporotic subjects. The best cut point to determine the discordance was estimated for women and men separately.

All statistical analyses were performed with STATA Statistical Software Release 14.0 (StataCorp. 2015. Stata Statistical Software: Release 14. College Station, TX: StataCorp LP). *P*-values less than 0.05 were considered to be statistically significant.

## Results

**Fig. 1:**
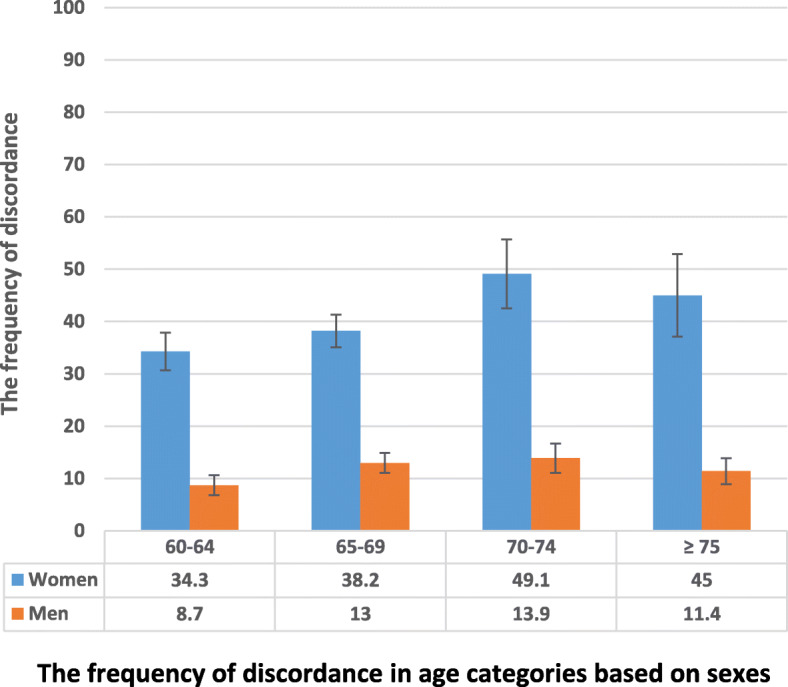
The frequency of discordance in non-osteoporotic elderly women and men in different age groups. Discordance is defined as TBS worse than BMD. Error bars present standard error

The distribution of 2283 elderly including 1142 women and 1141 men (BMI range of 15 to 37 Kg/m^2^) in the BMD and TBS categories is presented in Table 1. After the exclusion of 948 participants with osteoporosis, 1335 non-osteoporotic elderly women and men were included in the study for further analysis. Non-osteoporotic individuals were categorized as discordant and others, as described in Methods and shown in Table 1. Discordance was observed in 112 (24.5%) of 457 non-osteoporotic women, and 54 (6.2%) of 878 non-osteoporotic men. Discordance is more frequently observed in elderly men and women aged 70 to 74 years as illustrated in Fig. 1.

**Table 1:** Distribution of the Study Population in Different BMD and TBS Categories

			All sites BMD		
			1. Normal	2. Osteopenia	3. Osteoporosis
TBS	Women	1. Normal	28 (66.7%) ^b^	132 (31.8%) ^b^	84 (12.3%) ^c^
		2. Partially degraded	12 (28.6%) ^a^	185 (44.6%) ^b^	211 (30.8%) ^c^
		3. Degraded	2 (4.8%) ^a^	98 (23.6%) ^a^	390 (56.9%) ^c^
	Men	1. Normal	239 (93.4%) ^b^	453 (72.8%) ^b^	116 (44.1%) ^c^
		2. Partially degraded	14 (5.5%) ^a^	132 (21.2%) ^b^	87 (33.1%) ^c^
		3. Degraded	3 (1.2%) ^a^	37 (6.0%) ^a^	60 (22.8%) ^c^

BMD: Bone Mineral Density; TBS: Trabecular Bone Score; BMD categorized based on the lowest T-score of lumbar spine, femoral neck and total hip; Normal: lowest T-score ≥ −1.0; Osteopenia: −2.5 < lowest T-score < − 1.0; Osteoporosis: Lowest T-score ≤ − 2.5; TBS: Normal TBS > 1.31; Partially degraded: TBS 1.23–1.31; Degraded: TBS < 1.23; a: Discordant Subjects defined as “non-osteoporotic individuals with TBS worse than BMD” (including subjects with degraded TBS/normal BMD, degraded TBS/osteopenia, and partially degraded TBS/normal BMD); b: Others (including subjects with normal BMD/normal TBS, osteopenia/normal TBS, and osteopenia/partially degraded TBS); c: Excluded subjects with osteoporosis based on BMD of all sites

The characteristics and comparison of the study population in each group stratified by sex are shown in Table 2. WC, BMI, and smoking were statistically significantly higher in the discordant men and women compared to the others. Also, in discordant women, age and years since menopause were significantly higher compared to the others.

**Table 2:** Comparison of Characteristics Between Non-Osteoporotic Subjects with Discordance (TBS Worse than BMD) and Others

		Women (*N* = 457)			Men (*N* = 878)		
		Discordant ***N*** = 112	Others ***N*** = 345	***P*** value	Discordant ***N*** = 54	Others ***N*** = 824	*P* value
**Continuous variables (Mean ± SD)**							
Age (year)		67.9 (5.0)	66.8 (4.7)	**0.030**	69.1 (5.8)	69.0 (6.0)	0.888
Years since menopause		20.4 (7.7)	18.8 (7.3)	**0.046**	–	–	–
Anthropometric measures	Height (cm)	153.9 (5.7)	154.5 (5.5)	0.268	166.2 (6.1)	166.7 (6.1)	0.541
	Weight (kg)	72.3 (9.9)	69.6 (9.7)	**0.013**	84.3 (10.9)	73.5 (10.9)	**< 0.001**
	BMI (kg/m^2^)	30.5 (3.6)	29.1 (3.7)	**< 0.001**	30.5 (3.4)	26.4 (3.4)	**< 0.001**
	WC (cm)	106.6 (9.2)	101.0 (10.0)	**< 0.001**	109.5 (9.2)	97.6 (9.7)	**< 0.001**
Lipid profile	TC (mg/dl)	196 (48)	192 (47)	0.463	176 (38)	173 (41)	0.652
	TG ^a^ (mg/dl)	137 (90)	135 (78)	0.418	122 (62)	118 (75)	0.502
	LDL-C (mg/dl)	117 (42)	115 (42)	0.586	103 (34)	104 (35)	0.770
	HDL-C (mg/dl)	49 (11)	47 (11)	0.242	44 (13)	42 (10)	0.167
Creatinine ^a^ (mg/dl)		0.93 (0.16)	0.93 (0.2)	0.842	1.21 (0.32)	1.16 (0.28)	0.203
**Categorical variables, n (%)**							
Diabetes (yes)		50 (44.6%)	133 (38.6%)	0.253	21 (38.9%)	274 (33.2%)	0.396
Rheumatoid arthritis (yes)		5 (4.5%)	7 (2.0%)	0.165	0	1 (0.1%)	0.798
Underlying Diseases ^b^		6 (5.4%)	17 (4.9%)	0.857	2 (3.7%)	28 (3.4%)	0.905
Fracture History ^c^		18 (16.2%)	50 (14.5%)	0.657	1 (1.8%)	31 (3.8%)	0.466
Supplements	Vitamin D	14 (12.5%)	47 (13.6%)	0.761	2 (3.7%)	31 (3.8%)	0.983
	Calcium	12 (10.7%)	52 (15.1%)	0.248	2 (3.7%)	26 (3.2%)	0.824
Calcium intake	High	5 (4.6%)	16 (4.6%)		4 (7.4%)	83 (10.2%)	
	Moderate	33 (30.0%)	130 (37.8%)		30 (55.6%)	404 (49.4%)	
	Low	72 (65.4%)	198 (57.6%)	0.319	20 (37.0%)	330 (40.4%)	0.636
Physical activity (Yes)		25 (22.3%)	103 (29.9%)	0.123	13 (24.1%)	201 (24.4%)	0.958
Smoking	No	47 (42.0%)	195 (56.5%)		14 (25.9%)	373 (45.3%)	
	Past	41 (36.6%)	90 (26.1%)		26 (48.2%)	290 (35.2%)	
	Current	24 (21.4%)	60 (17.4%)	0.025	14 (25.9%)	161 (19.5%)	0.021

BMI: Body Mass Index; WC: Waist Circumference, TG: Triglyceride; LDL-C: Low-density lipoprotein-Cholesterol; HDL-C: High-density lipoprotein-Cholesterol; TC: Total Cholesterol; TBS: Trabecular Bone Score; BMD: Bone Mineral Density; a. Median (IQR) b. Including rheumatoid arthritis, chronic kidney disease, liver disease, seizure, hyperthyroidism; c. Fractures after 45 years with minor trauma; Calcium intake: high > 1000 mg/day, moderate 500–1000 mg/day, and low < 500 mg/day

In univariable analysis, discordance in women was significantly associated with age (OR: 1.05 (1.00–1.09)), years since menopause (OR: 1.03 (1.00–1.06)), BMI (OR: 1.10 (1.04–1.17)), WC (OR: 1.07 (1.04–1.10)), and past smoking (OR: 1.89 (1.16–3.08)) (Table 3). However, in multivariable analysis, after adjusting for possible confounders, the association remained significant for years since menopause (OR: 1.04 (1.00–1.07)) and WC (OR: 1.09 (1.05–1.14)).

**Table 3:** Univariable And Multivariable Regression Analysis of Non-Osteoporotic Elderly Individuals to Identify Factors Associated with Discordance

		Women		Men	
		Univariable model	Multivariable Model	Univariable model	Multivariable Model
		OR (95%CI)	OR (95%CI)	OR (95%CI)	OR (95%CI)
Age		1.05 (1.00–1.09) ^a^		1.00 (0.96–1.05)	–
Years since menopause		1.03 (1.00–1.06) ^a^	**1.04 (1.00–1.07)** ^a^		
BMI		1.10 (1.04–1.17) ^a^	0.96 (0.87–1.06)	1.39 (1.27–1.51) ^b^	1.12 (0.94–1.34)
Waist circumference		1.07 (1.04–1.10) ^b^	**1.09 (1.05–1.14)** ^b^	1.14 (1.10–1.18) ^b^	**1.10 (1.03–1.17)** ^a^
Fracture History		1.14 (0.63–2.05)	–	0.48 (0.06–3.59)	–
Smoking	Past	1.89 (1.16–3.08) ^a^	1.35 (0.78–2.35)	2.39 (1.23–4.66) ^a^	2.02 (0.99–4.10)
	Current	1.66 (0.94–2.94)	1.75 (0.94–3.26)	2.32 (1.08–4.97) ^a^	**2.54 (1.10–5.84)** ^a^
Glucose		1.00 (1.00–1.01)	–	1.00 (1.00–1.01)	–
Diabetes		1.29 (0.84–1.98)	–	1.28 (0.73–2.25)	–
Physical activity ^d^		0.68 (0.41–1.11)	0.79 (0.46–1.37)	0.98 (0.52–1.87)	–
TC		1.00 (1.00–1.01)	–	1.00 (0.99–1.01)	–
TG		1.00 (0.99–1.00)	–	1.00 (0.99–1.00)	–
LDL-C		1.00 (1.00–1.01)	–	1.00 (0.99–1.01)	–
HDL-C		1.01 (0.99–1.02)	–	1.02 (1.00–1.05)	**1.03 (1.00–1.06)** ^a^
Vitamin D supplement		0.91 (0.48–1.72)	–	0.98 (0.23–4.22)	–
Calcium Supplement		0.68 (0.35–1.32)	–	1.18 (0.27–5.11)	–
Calcium intake	Moderate	0.81 (0.28–2.38)	–	1.54 (0.53–4.49)	–
	Low	1.16 (0.41–3.29)	–	1.26 (0.42–3.78)	–

Discordance is defined as TBS Worse Than BMD. Odds ratios with 95% confidence intervals are presented. BMI: Body Mass Index; TG: Triglyceride; LDL-C: Low density lipoprotein-Cholesterol; HDL-C: High density lipoprotein-Cholesterol; TC: Total Cholesterol; TBS: Trabecular Bone Score; BMD: Bone Mineral Density; Calcium intake: high > 1000 mg/day, moderate 500–1000 mg/day, and low < 500 mg/day; a. *P*-value < 0.05, b. P-value < 0.001; We used low physical activity as reference value. We used high calcium intake as the reference value. To reduce collinearity, the multivariable analysis was performed after exclusion of BMI and no significant change in results was observed

As shown in Table 3, in men, discordance was significantly associated with BMI (OR: 1.39 (1.27–1.51)), WC (OR: 1.14 (1.10–1.18)), past smoking (OR: 2.39 (1.23–4.66)), and current smoking (OR: 2.32 (1.08–4.97)). In multivariable analysis, we showed that WC was positively associated with discordance (OR: 1.10 (1.03–1.17)), which means that each centimeter increase in WC may lead to a 10% increase in the likelihood of discordance. We also found that current smoking (OR: 2.54 (1.10–5.84)) and HDL-C (OR: 1.03 (1.00–1.06)) are significantly associated with discordance in the non-osteoporotic elderly men.

**Fig. 2:**
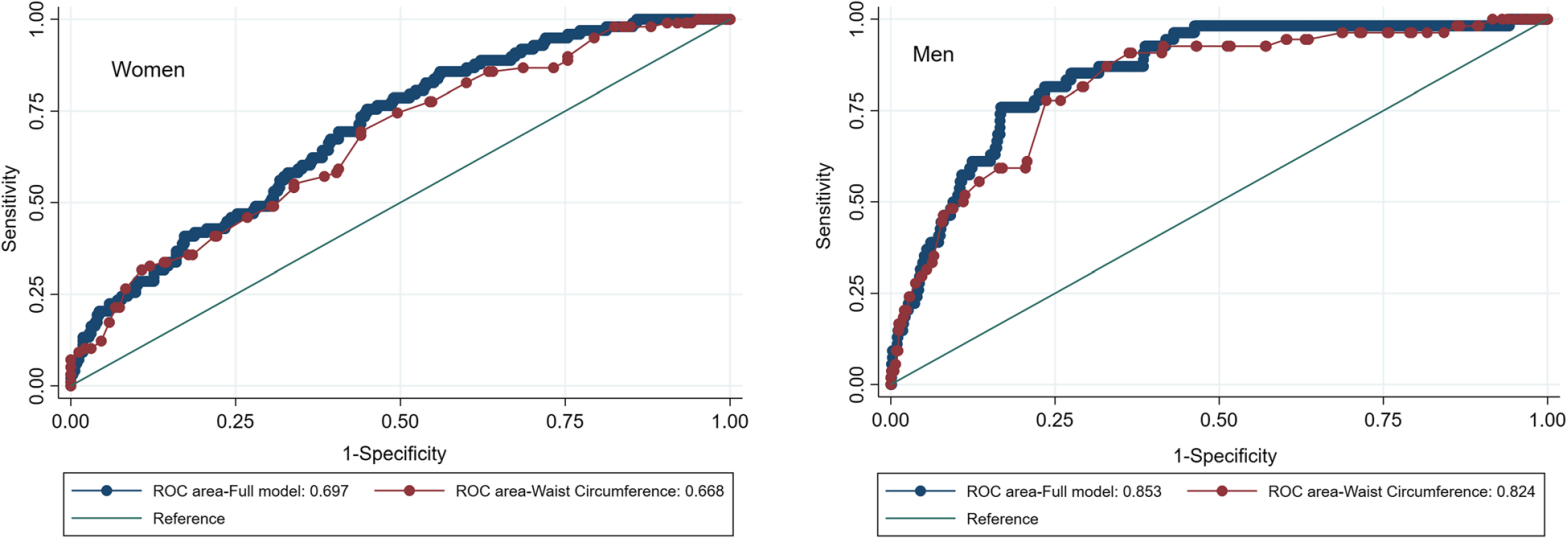
ROC curve in predicting discordance in non-osteoporotic elderly women and men. Discordance is defined as TBS worse than BMD

The ROC curves showed a good performance of the developed models with the AUC of 0.853 (95%CI: 0.806–0.899) and 0.697 (95%CI: 0.641–0.753) in men and women, respectively. In men, the AUC of the model with WC as the single variable was 0.824 (95%CI: 0.768–0.879) compared with AUC of 0.853 for the full model; in women the AUCs were 0.668 (95%CI: 0.608–0.728) and 0.697 respectively, presenting the major role of WC as a predictor of discordance as shown in Fig. 2. The optimal cut-off points for WC to is 102.25 cm (Sen: 0.68, Spc:0.56) in women and 104.5 cm (Sen: 0.78, Spc:0.76) in men.

## Discussion

After excluding the osteoporotic subjects, we categorized the remainders, into two groups of discordant defined as “TBS worse than BMD” and others, and evaluated the factors associated with discordance. We found that 24.5 and 6.2% of the non-osteoporotic elderly women and men, respectively, had a more degraded TBS than BMD. Among the various clinical factors evaluated, this condition was associated with WC in both women and men, with years since menopause in women and with current smoking and HDL-C in men.

In a recent study on 1505 Korean women older than 40 years, 10.6% of participants had more degraded TBS than BMD, and they were older, heavier, with higher BMIs [8]; this finding is comparable to our overall observation of 11.9% of elderly women (osteoporotic and non-osteoporotic according to BMD) with TBS worse than BMD.

Menopause is associated with bone loss due to rapid estrogen withdrawal. After menopause, LS BMD and TBS values decrease and the risk of fracture increases with age. Degenerative disease increases with age and affects LS BMD. BMD measurement has certain limitations in advanced age and maybe falsely increased by artifacts due to degenerative disease, like osteoarthritis. We found that years since menopause was associated with TBS worse than BMD. LS BMD increased after 62.5 years of age in a study including 1500 women from the OsteoLaus cohort, while the TBS was not affected by degenerative changes and continued to decline continuously and steadily with age [27]. Likewise, TBS was shown to be numerically superior to BMD measured at any site concerning fracture risk prediction in another study in elderly community-dwelling women from a sample of the Swiss population [28]. Therefore, TBS plays a leading role in the diagnosis in complement to BMD especially in the elderly.

We observed that BMI and WC were higher in the discordant group compared to the others, in men and women. Likewise, BMI was higher in Korean women with TBS worse than BMD [8]. WC, but not BMI remained significantly associated with “TBS worse than BMD” after adjustment for different potential confounders. Similarly, abdominal fat accumulation negatively affected TBS values, independent of the lumbar spine BMD, and WC had a greater influence on TBS values than BMI in a sample of overweight/obese men [18]. While total body fat mass was associated with lumbar spine BMD, android fat and visceral fat were inversely associated with TBS [19]. Besides, several studies have shown a positive relationship between BMI and BMD [29, 30] and a negative correlation between BMI and TBS [30, 31]. The negative correlation reported frequently between TBS and BMI or weight could be due to the negative effect of excess visceral fat on bone quality by negatively affecting bone microarchitecture, or could be a technical limitation of TBS. Regional soft tissue by attenuating X-rays may have a noise effect on TBS leading to underestimated values [32]. The issue is more observed in the current TBS software (TBS iNsight®) which is based on algorithms accounting for BMI as a surrogate for soft tissue thickness. A more recent version of the TBS software (version 4.0), based on an algorithm that takes into account the regional soft tissue thickness itself instead of BMI, has claimed to be free from the previously recognized technical limitation of TBS and showed to have overcome the debatable residual negative correlation of the current TBS with body size and BMI [33]; however, this version is not yet commercially available. Altogether, our findings along with others, suggest that we should focus on TBS values especially in those with higher WC, regardless of BMD.

We found that current smoking (OR: 2.54) in the men, were an independent factor associated with discordance. A recent study observed abnormal TBS in a significant proportion (40%) of the smokers with and without chronic obstructive pulmonary disease, and BMI, age, number of exacerbations, and the degree of airway obstruction were the predicting factors. The abnormal TBS was not exactly concordant with the ones that had abnormal BMD values [17]. This finding, in line with ours, emphasizes the value of TBS when evaluating smokers at risk of fragility fractures with normal BMD or osteopenia.

Although HDL-C did not differ significantly between the discordant group and others, the association was remained in multivariable analysis after adjustment for possible confounders in non-osteoporotic men (OR: 1.03). As reported previously, in the study population, HDL-C was negatively correlated to the TBS in elderly men (beta: 0.083) but not in women, after adjusting for several confounders [34].

The present study has some limitations. First, we did not have access to some data such as the vitamin D level of participants. Second, we did not perform vertebral exclusion if there were more than 1.0 T-score difference between adjacent vertebrae. Third, we could not evaluate the effect of different comorbidities such as chronic kidney disease and hyperparathyroidism on the discordance due to insufficient data and low prevalence of the comorbidities. Finally, we used Hologic densitometers, and the effects of BMI on TBS seem to be greater with Hologic densitometers than GE densitometers [35]. However, the strength of this study lies in the fact that we described the distribution of baseline BMD and TBS categories in a large sample size population-based study of Iranian elderly men as well as women. In addition, we determined factors associated with discordance between TBS and BMD in non-osteoporotic elderly women and men. Besides, we used conventional and accessible risk factors and performed multivariable regression analysis considering several potential confounders including age, years since menopause, BMI, WC, lipid profile, diabetes, smoking status, physical activity, use of supplements, and calcium intake. However, no causal relationship can be concluded due to the cross-sectional design of the study. Further studies are required to confirm the findings.

## Conclusion

This study showed that years since menopause and WC were associated with a worse TBS than BMD in the non-osteoporotic elderly women, and WC, current smoking, and HDL-C were associated with worse TBS than BMD in the non-osteoporotic elderly men. Our findings underscore the complementary role of TBS in practice for the assessment of bone health and suggest that we should focus on TBS values in the elderly individuals, especially those with higher WC or with a history of smoking, who seem to be more likely to have more degraded TBS and increased risk of fragility fracture despite better BMD status.

## Data Availability

The datasets used and/or analyzed during the current study are available from the corresponding author on reasonable request.

## Abbreviations

AUC: area under a ROC curve
BMD: Bone mineral density
BMI: body mass index
DXA: dual-energy X-ray absorptiometry
FRAX: fracture risk assessment tool
HDL-C: high-density lipoprotein-cholesterol
LDL-C: low-density lipoprotein-cholesterol
ROC: receiver operating characteristic
TBS: trabecular bone score
TC: total cholesterol
TG: triglyceride
WC: waist circumference
